# The Promise of an Evolutionary Perspective of Alcohol Consumption

**DOI:** 10.1177/26331055231163589

**Published:** 2023-04-04

**Authors:** Benjamin L Clites, Hans A Hofmann, Jonathan T Pierce

**Affiliations:** 1 Department of Neuroscience, University of Texas at Austin, Austin, TX, USA; 2 Waggoner Center for Alcohol and Addiction Research, University of Texas at Austin, Austin, TX, USA; 3 Institute for Cellular & Molecular Biology, University of Texas at Austin, Austin, TX, USA; 4 Institute for Neuroscience, University of Texas at Austin, Austin, TX, USA; 5 Department of Integrative Biology, University of Texas at Austin, Austin, TX, USA

**Keywords:** Ethanol, genetic variation, selection, comparative approach

## Abstract

The urgent need for medical treatments of alcohol use disorders has motivated the search for novel molecular targets of alcohol response. Most studies exploit the strengths of lab animals without considering how these and other species may have adapted to respond to alcohol in an ecological context. Here, we provide an evolutionary perspective on the molecular and genetic underpinnings of alcohol consumption by reviewing evidence that alcohol metabolic enzymes have undergone adaptive evolution at 2 evolutionary junctures: first, to enable alcohol consumption accompanying the advent of a frugivorous diet in a primate ancestor, and second, to decrease the likelihood of excessive alcohol consumption concurrent with the spread of agriculture and fermentation in East Asia. By similarly considering how diverse vertebrate and invertebrate species have undergone natural selection for alcohol responses, novel conserved molecular targets of alcohol are likely be discovered that may represent promising therapeutic targets.

## Introduction

Alcohol use disorder (AUD) is one of the most common psychiatric diseases in the US affecting more than 1 in 10 American adults.^1^ Globally, the World Health Organization estimates nearly 6% of deaths and 5% of injury burden can be attributed to alcohol abuse.^2^ Despite the magnitude of the damage that alcohol abuse causes, there are relatively few treatment options available.^3^ One approach to identify viable treatments has focused on studying molecular genetic contributions for AUD. A genetic approach is promising because twin and adoption studies estimate that about half of the risk for alcohol dependence is heritable.^4^ Thus, identifying these genetic factors that underlie AUD may lead to sorely needed novel treatments, while also providing insights into the basic biology of AUD. Candidate targets for treatments may be identified by searching for specific genetic variants associated with molecules that contribute to population-wide differences in AUD risk.^5^ Early g–enome-wide association (GWA) studies on individual variation in AUD risk identified only a few replicable associations in human populations, most notably genes involved in the metabolism of alcohol (for review see Tawa et al^6^). However, recent GWA efforts have used expanded sample sizes and genomic resources that cross multiple populations to identify promising new candidate genes, as well as shedding light on the shared architecture of alcohol abuse and other psychiatric traits.^7,8^ Even still, GWA studies on human populations cannot be causally validated, and often end with correlations. Novel population genetic strategies are needed to identify additional genetic effectors of alcohol response.

## An Ethological Perspective of Alcohol Use

Ethanol presents both an ecological challenge as well as an opportunity to a wide array of species across taxa and time. While ethanol is toxic when consumed to excess,^9^ it can also serve as a volatile signal to locate calorie-rich food sources (eg, fruit patches containing rotting fruit),^10-12^ or potentiate pheromone signaling when searching for potential mates.^13^ For those organisms that have adapted to exploit it, ethanol also represents a source of calories in and of itself, especially in impoverished conditions.^14-16^ The ethanol-induced impairment of behaviors critical for evolutionary fitness displays natural variation (eg, male mating success of the fruit fly *Drosophila melanogaster*^17^). Understanding the evolutionary relationships between alcohol and the variety of species that have evolved to exploit it will expand our view of alcohol, and its effects on humans today, and point to novel ways to identify conserved molecular targets of alcohol response.

In the early 2000s, alongside the emerging field of evolutionary medicine,^18^ some asked whether the cross-cultural phenomenon of alcoholism could be attributed to an “evolutionary mismatch.”^19^ This idea posited that some traits, which were adaptive in the ancestral environment, become deleterious when “mismatched” to the modern environment.^20^ Lieberman,^21^ for example, speculated that in our supposedly resource-scarce ancestral environment it was beneficial to crave and consume high-sugar foods, as they were rare and high in calories. Others hazarded that human contact with alcohol began with the advent of agriculture and fermentation some 9000 years ago.^22^ In modern industrialized society, where sugary foods are ubiquitous and cheap, those same traits may then lead some individuals to consume sugars to the point of chronic illness (eg, diabetes and obesity).^23,24^ A similar hypothesis has been proposed regarding ethanol. Chronic but low-level consumption of ethanol may have been advantageous for health and fitness in an ancestor, but when these behavioral and physiological adaptations met a society where highly concentrated alcohol became easily accessible, a mismatch occurred, and the “evolutionary hangover” began.^25-28^ However, given new evidence accumulated over the last 2 decades, there is a need to reevaluate the behavioral ecology of alcohol consumption and its potentially long history with the human lineage.

## Frugivores and Alcohol Consumption

**Figure 1.**
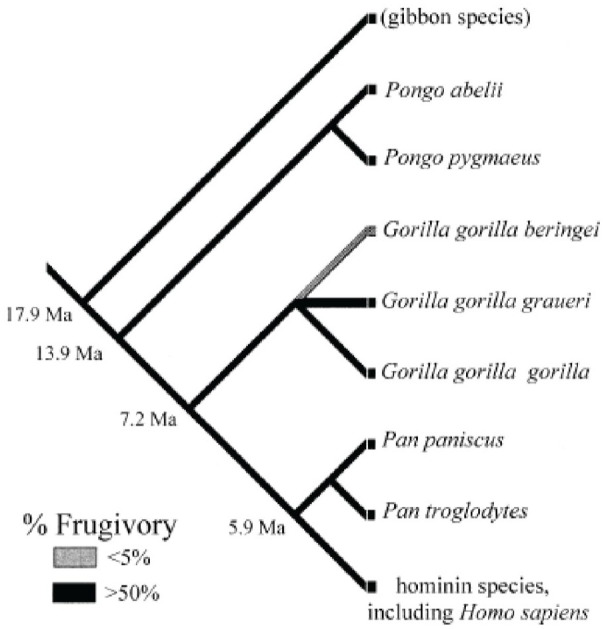
Phylogeny of extant hominoid species. Branches are gray-scale coded by % fruit in average diet for each species. Ticked fills represent uncertainty with respect to dietary fruit contribution to the diet of ancestral hominine species. Source: From Dudley.^26^

An evolutionary perspective of alcohol abuse based on evidence must first acknowledge that our hominoid ancestors, who consumed ripe fruits, ingested alcohol at low levels already ~24 million years ago,^26^ which may have provided ample opportunity for adaptation to occur (Figure 1). The hominid transition to terrestrial foraging some 10 to 20 MYA^28^ may have accelerated this process as the consumption of low-levels of alcohol via overripe and rotting fruits encountered on the ground may have become more likely. Independent of the ultimate cause, several mechanisms for realizing a fitness benefit have been proposed. One hypothesis posits that natural selection favored primates attracted to alcohol, even if the benefits of this attraction were indirect. For example, volatile ethanol molecules emanating from a piece of fermenting fruit might act as a sensory cue used to locate a food patch,^10,11,29^ or as an appetite stimulant, an effect demonstrated in a number of species including modern humans.^30,31^ Others contend that the direct caloric content of alcohol provides a fitness benefit to those that can exploit those calories whilst minimizing the toxic effects of alcohol consumption.^9,14^ Still, there remains a dearth of data on the alcohol content of wild fruits at different stages of ripeness or rot. Dudley^27,32^ assayed wild Panamanian Palm fruits and found them to contain average levels of about 0.56% ± 1.04% v/v alcohol, with some overripe fruit samples containing up to 5% alcohol (about the content of typical beers).

Despite earlier claims to the contrary, many recent studies find that frugivores *do* prefer overripe and rotting fruits, whilst others have observed the direct consumption of alcoholic solutions. For example, Peris et al^29^ looked at the dietary habits of wild seed disperser and pulp feeding species across 2 biomes and found that rotting fruits inoculated with *Penicillium digitatum* fungus were overwhelmingly preferred by local frugivores. Others found that African elephants (*Loxodonta africana*) could identify fruit sugar content based on scent alone, with volatile ethanol in the scent plume accounting for nearly 50% of the variance in which fruits were preferred.^33^ Similarly, a randomized 5-choice test on 2 nectar-feeding primates, the slow loris (*Nycticebus coucang*) and aye-aye (*Daubentonia madagascariensis*) found that both species prefer higher ethanol concentrations (3% and 5%) over lower concentrations (0% and 1%).^34^ Strikingly, Hockings et al^35^ reported that wild West African chimpanzees consume alcoholic palm nectar (3.1%-6.7% v/v ethanol) repeatedly over a period 17 years. These observations suggest that incidental or voluntary alcohol consumption in our frugivorous ancestors is more plausible than was previously thought.^36-38^

## Evidence of Molecular Adaptations to Alcohol Metabolism Amongst Frugivores

Frugivory is common across animals, so we might ask whether diverse fruit-eating species share molecular adaptations to alcohol metabolism. Across species, alcohol is first metabolized by alcohol dehydrogenase (ADH), producing a toxic intermediate, acetaldehyde, which is in turn converted to harmless acetate by the enzyme aldehyde dehydrogenase (ALDH) (for a more complete review of alcohol metabolic genes, see Oota et al^39^). Interestingly, in *D. melanogaster*, increased alcohol metabolism correlates with ethanol content of species-specific food niches,^40^ and intra-specific variation in alcohol sensitivity correlates with ADH activity toward alcohol in *D. melanogaster*.^41^ A similar pattern has been found in birds: ADH enzymes of passerines with higher proportions of fruit in their diets show increased capacity to metabolize alcohol.^42^ A recent study by Janiak et al^43^ used comparative genomics to analyze the relationship between dietary niche and alcohol metabolism. They found that the fraction of the diet that is plant-based significantly correlated with *ADH7* pseudogenization across 79 mammal species. Thus, some adaptations toward alcohol appear to either be conserved across a wide range of tropical frugivorous species or have evolved convergently.

## Evidence of Adaptation to Alcohol Metabolism in Great Apes

**Figure 2.**
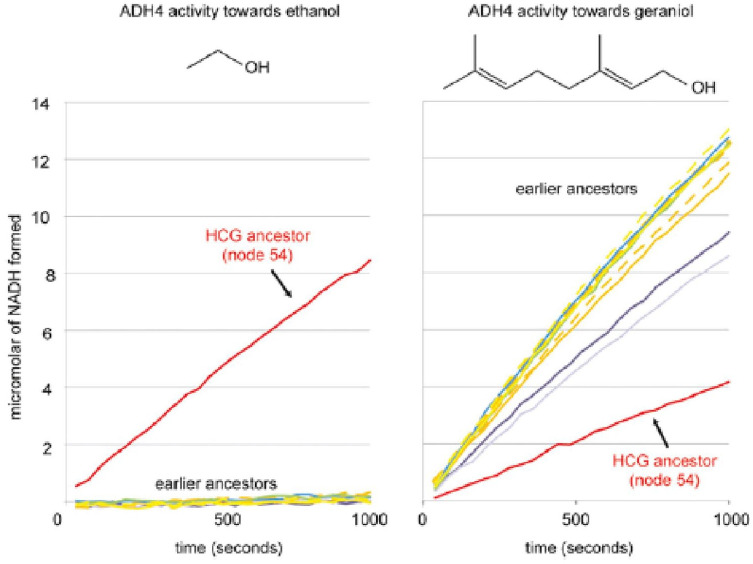
Neofunctionalization of hominid Alcohol Dehydrogenase 4 (*ADH4*) toward alcohol. *ADH4* genes of extant and ancestral primates were synthesized and assayed for activity against a variety of substrates. A variant in ADH4 (A294V) that arose in the last common ancestor of the great apes shifted activity of the enzyme away from common plant terpenoids toward ethanol. Source: From Carrigan et al.^44^

Recent research has also provided evidence that the consumption of fermented fruit was accompanied by adaptive evolution of genes involved in alcohol metabolism in great apes. For example, Carrigan et al^44^ assayed enzyme activity of *ADH4* genes from across the primate clade and found a single amino acid variant that arose in the last common ancestor of chimpanzees, gorillas, and humans (Figure 2). This variant causes high activity toward ethanol, in contrast to the other primate ADH4 proteins, which show low activity toward ethanol, but high activity toward anti-feedant terpenoids, such as geraniol, commonly found in leafy plants. This novel variant appears to have arisen ~10 MYA, around the time when our ancestors transitioned to terrestrial foraging, which may have led to the consumption of overripe and rotting fruits on the ground. Interestingly, the only other primate species that harbored this variant was the aye-aye, which prefers the higher concentration of alcohol offered in a 2-choice test.^34^

## Evidence of Adaptation to Alcohol Metabolism in Modern Humans

The alcohol metabolic pathway presents also the best evidence of recent human adaptations toward alcohol consumption. Studies on the numerous *ADH* and *ALDH* genes provide perhaps the most compelling evidence that humans have undergone recent evolution with respect to alcohol consumption. These genes vary within and between populations, and allelic variation correlates strongly with AUD risk. Variants that either increase ADH activity or decrease ALDH activity cause build-up of toxic acetaldehyde which quickly causes facial flushing, tachycardia, nausea, that together serve as a deterrent to drinking.^45^ These variants are more common in East Asian populations than they are in European, African, or North American populations, and these differences correlate with markedly lower rates of alcoholism (for review see Edenberg^46^). These loci also show signs of recent selection in East Asia,^47-50^ suggesting that these patterns are not merely consequences of genetic drift.

**Figure 3.**
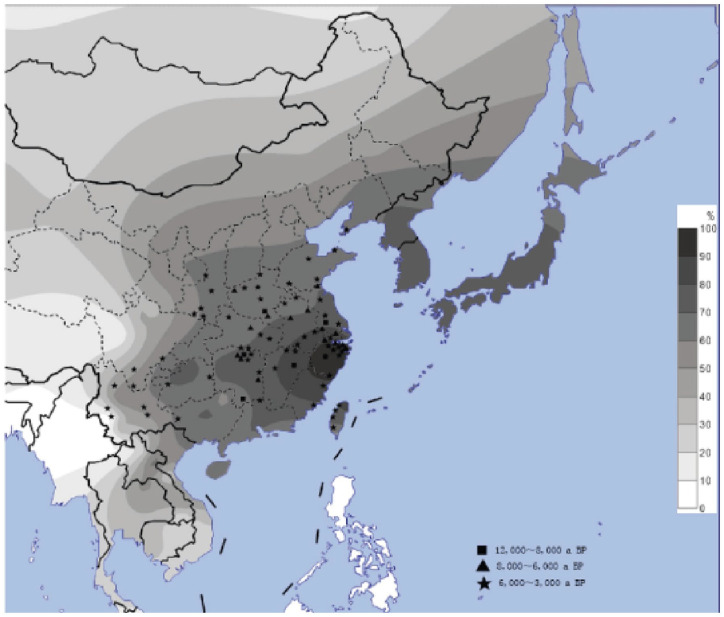
Distribution of *ADH1B* rs1229984 variant frequency in East Asia. Darker shades indicate higher allele frequency of *ADH1B* rs1229984, a single nucleotide polymorphism associated with lowered risk of alcoholism. Archeological sites of Neolithic rice cultivation are marked by squares, triangles, and stars, where each shape represents age of site from oldest to youngest respectively. Source: From Peng et al.^47^

A closer look at the variation within Asian populations provides even more evidence for recent adaptation in alcohol metabolism after the advent of fermentation subsequent to the introduction of agriculture. Peng et al^47^ found that the *ADH1B* rs1229984 variant, which results in a ADH1BArg47His polymorphism and is protective against alcoholism, becomes less frequent in an east to west gradient, with contemporary populations ranging from 98.5% allele frequency in south-east China to only 2% in south-west China (Figure 3). The *ADH1B* rs1229984 variant represents a gain-of-function allele that increases production of acetaldehyde. Intriguingly, this pattern mirrors the pattern of early agriculture and fermentation, which first appeared in the southeast (8000-12 000 years ago) before spreading west (3000-6000 years ago). A separate study directly tracked the allelic expansion of the rs1229984 variant in northern China across time by genotyping ancient remains dated from between 2,500 BC and 220 AD. They found that a marker of rs1229984 allele increased rapidly over the last 4000 years, suggesting temporal and geographical bounds on a putative selective mechanism.^51^ These data provide persuasive evidence that *Homo sapiens* underwent recent selection with respect to alcohol consumption, at least in Southeast Asia, although alternative selective scenarios that predate the invention of fermentation—such as toxins produced by fungi found on moldy rice or infectious disease—have been suggested.^52^ Other studies,^53-55^ including a recent well-powered GWA study of alcohol-use traits,^56^ indicate that *ADH1B* underwent a similar selection process in Africa. A distinct *ADH1B* gain-of-function Arg369Cys variant, rs2066702, found in African-American and some Native American populations was found to correlate with lower incidence of alcoholism, lower maximal habitual alcohol intake, and less problematic alcohol use. More recent GWA studies have confirmed the highly significant association of variants in ADH1B with alcohol consumption traits across African, Asian, and European populations. For instance, recent GWA studies leveraging millions of subjects identified 11 conditionally independently variants in ADH1B that associated with alcohol consumption.^57,58^

Interestingly, the adaptive genetic variation in alcohol metabolism found in humans is already the target of one of 3 currently approved pharmaceutical interventions to treat AUD.^59^ Specifically, the drug Disulfiram acts by interfering with ALDH activity. When administration is supervised, Disulfiram pharmacologically confers protection against AUD to a degree that resembles that of Japanese individuals who are homozygous for the hypomorphic variant in ALDH.^60^ Left unsupervised, however, patients often fail to adhere to Disulfiram treatment and risk relapse of alcohol consumption.^59^ Modern AUD treatments therefore aim to target candidate physiological and brain mechanisms that are thought to underlie maladaptive patterns of alcohol consumption.^61^

## Sex Differences in Alcohol Metabolism Are Widespread

It is unlikely, however, that shared natural genetic adaptations toward alcohol consumption amongst frugivores are limited to its metabolism. Conserved sex differences provide another example. In *H. sapiens*, males are less sensitive to alcohol consumption and have higher rates of alcoholism than females.^62^ In the long-tailed macaque (*Macaca fascicularis*), a primate model that shares frugivory with humans, males are also more likely than females to voluntarily consume alcohol and to maintain high consumption, at least in a lab setting.^63^ Similarly, *D. melanogaster* males show higher ethanol hyperactivity and resistance to sedation than do females.^64^ By contrast, studies in Long-Evans rats find the opposite effect.^65^ While these sex differences have some basis in differential metabolism, there are likely other shared mechanisms that explain this pattern as well. Thus, frugivorous species may be better suited as model systems for elucidating the antecedent causes of individual differences in alcohol consumption (eg, genetic bases of attraction to alcohol, sex differences in alcohol phenotypes, etc.) than their non-frugivorous counterparts, such as rodents.

## Beyond Metabolism: Conserved Molecular Pathways Regulate Alcohol Sensitivity Across Diverse Species

In addition to the variants in ethanol metabolic enzymes discussed above that appear to have been under selection over the course of our evolutionary history, recent GWA studies have identified candidate genes that are significantly associated with alcohol use traits, have no known relation to ethanol metabolism, and have yet to be examined from a molecular evolution perspective. These studies have provided evidence for a wealth alcohol-related genes by leveraging enormous sample sizes (0.5-3.4 million subjects per study) as well as more careful selection criteria and phenotyping.^58,66,67^ Reassuringly, a number of these genes already have experimental evidence suggesting causative effects on alcohol phenotypes. For instance, Liu et al^57^ identified 3 variants that implicate 2 genes in both nicotine and alcohol addiction, namely phosphodiesterase 4B (PDE4B), which plays a role in cellular signal transduction by regulating the cellular concentrations of cAMP, and cullin 3 (CUL3), which mediates the response to the steroid aldosterone (which is thought to modulate alcohol consumption).^68,69^ Early studies on drugs that target PDE4B are producing promising results to reduce alcohol consumption in rodents and, impressively, even in patients.^70,71^ In addition, these GWA studies also implicated genes that are part of the glutamate ionotropic receptor kainate type subunit 2 (GRIK2) protein-protein interaction subnetwork, suggesting another promising entry into studying the brain mechanisms underlying AUD.^57^ Many other genes function in glucose and carbohydrate processing, leading the authors to hypothesize that variation in caloric processing influences alcohol consumption.^57^ One intriguing pair of genes discovered included urocortin and its receptor, the corticotropin-releasing hormone receptor 1 (CRHR1).^57^ Corticotropin modulates stress hormone circuits, including cortisol, which are thought to be pivotally involved in withdrawal and relapse. Finally, the gene beta-Klotho, which was also identified in these human studies, was recently found to regulate FGF21-dependent preference to drink alcohol in mice.^72^

Most variants identified in these studies implicate genes without previously known relationships to alcohol phenotypes, even though their (often pleiotropic) effects on other phenotypes include immune or liver function. For instance, a variant of the zinc and manganese transporter SLC39A8 is associated with monocyte function in inflammation, glutamatergic neurotransmission, and metals homeostasis.^60^ And a variant in the serpin protease inhibitor A1 (SERPINA1) causes it to accumulate in the liver rather than move to the lungs, where it normally protects against toxins, raising risk of both lung and liver diseases.^73^ Taken together, these recent GWA studies provide exciting new targets for understanding how the human genome may have evolved in response to alcohol use by our ancestors.

Although rodents are widely used in alcohol research, the ethological relevance of alcohol consumption for several invertebrate model systems has provided excellent opportunities to discover evolutionarily conserved genetic effectors of alcohol response. For example, in the wild, the nematode *Caenorhabditis elegans* reproduces on rotting fruits which may contain low levels of alcohol.^74^*C. elegans* has been used for decades to study alcohol response in the lab. However, almost all research uses a single strain (N2) isolated nearly 50 years ago.^75^ Early studies using a more recently isolated wild Hawaiian strain of *C. elegans* (CB4856) discovered that natural variation in the neuropeptide Y receptor affects *C. elegans* alcohol response.^76^ Sequence variation in the neuropeptide Y receptor has also been implicated in variation in alcohol sensitivity in the fruit fly *D. melanogaster*,^77^ as well as AUD risk in human populations.^78,79^ Efforts to study natural variation in *D. melanogaster* have also identified genes with effects later demonstrated to be conserved in humans. Examples include DOPA decarboxylase, which is essential for the synthesis of amine neurotransmitters such as dopamine and serotonin,^80,81^ and the KCNQ family of potassium channels.^80,82^ These convergent lines of evidence suggest that humans have adapted to alcohol consumption, both in recorded history and in our more distant hominid past, and even in deep evolutionary time. In fact, comparative studies have convincingly demonstrated that similar molecular mechanisms underlying convergently evolved traits are more common than was previously believed,^83^ even across vast evolutionary distances and involving complex behavior (eg, aggression^84,85^; learned vocalizations^86^; monogamy^87^). This evolutionary framework suggests that, rather than standard isogenic lab strains, natural populations with alcohol consumption in their natural history, and in conjunction with GWA studies, comparative transcriptomics, and other emerging technologies, will uncover important new insights across diverse species.^88-90^
